# Cyclodextrin Inclusion Complex to Improve Physicochemical Properties of Herbicide Bentazon: Exploring Better Formulations

**DOI:** 10.1371/journal.pone.0041072

**Published:** 2012-08-27

**Authors:** Claudia Yáñez, Paulina Cañete-Rosales, Juan Pablo Castillo, Nicole Catalán, Tomás Undabeytia, Esmeralda Morillo

**Affiliations:** 1 Departamento de Química Orgánica y Fisicoquímica, Facultad de Ciencias Químicas y Farmacéuticas, Universidad de Chile, Santiago, Chile; 2 Instituto de Recursos Naturales y Agrobiología, Consejo Superior de Investigaciones Científicas, Sevilla, España; Università di Napoli Federico II, Italy

## Abstract

The knowledge of the host-guest complexes using cyclodextrins (CDs) has prompted an increase in the development of new formulations. The capacity of these organic host structures of including guest within their hydrophobic cavities, improves physicochemical properties of the guest. In the case of pesticides, several inclusion complexes with cyclodextrins have been reported. However, in order to explore rationally new pesticide formulations, it is essential to know the effect of cyclodextrins on the properties of guest molecules.

In this study, the inclusion complexes of bentazon (Btz) with native βCD and two derivatives, 2-hydroxypropyl-β-cyclodextrin (HPCD) and sulfobutylether-β-cyclodextrin (SBECD), were prepared by two methods: kneading and freeze-drying, and their characterization was investigated with different analytical techniques including Fourier transform infrared spectroscopy (FT-IR), differential thermal analysis (DTA), X-ray diffractometry (XRD) and differential pulse voltammetry (DPV). All these approaches indicate that Btz forms inclusion complexes with CDs in solution and in solid state, with a stoichiometry of 1∶1, although some of them are obtained in mixtures with free Btz. The calculated association constant of the Btz/HPCD complex by DPV was 244±19 M^−1^ being an intermediate value compared with those obtained with βCD and SBECD. The use of CDs significantly increases Btz photostability, and depending on the CDs, decreases the surface tension. The results indicated that bentazon forms inclusion complexes with CDs showing improved physicochemical properties compared to free bentazon indicating that CDs may serve as excipient in herbicide formulations.

## Introduction

Cyclodextrins (CDs) are cyclic oligosaccharides which provide an interesting organic host system, since they have a hydrophobic inner cavity available to form non-covalent host-guest inclusion complexes with a wide variety of organic molecules of appropriate shape and size [1]–[3]. This property has attracted considerable attention in the field of the encapsulation of molecules because some physicochemical properties of the guest can be notably changed by its inclusion. In this context, the pharmaceutical industry has successfully used this distinctive aspect of CDs in different pharmaceutical applications [4]. Some commercial formulations using CD are mentioned by Szejtli [5].

Nowadays, thanks to large improvements in cyclodextrin production and purification, the application of this organic host in another important area such as agriculture, is becoming a real possibility [6]. In agricultural production, the application of pesticides is of vital importance. However, excessive amount of pesticide is sometimes used to compensate that part of them is lost by different mechanism (evaporation, hydrolysis, inactivation by wetness or by contact with soil, degradation by light, etc). For this reason, an optimization in the herbicides formulations is necessary.

Molecular encapsulation of pesticides by CDs can enhance the efficacy and facilitate formulation of these agrochemicals in a variety of ways, for example, by increasing their flowability, wettability, dissolution rate, chemical and thermal stability, and reducing their volatility [7]. CDs have the capacity to increase the apparent solubility of several hydrophobic pesticides and thus, their bioavailability for biodegradation [8]–[10]. Besides, CDs present several advantages over solvents and nonionic surfactants including their lower toxicity and higher biodegradability [11].

**Figure 1:**
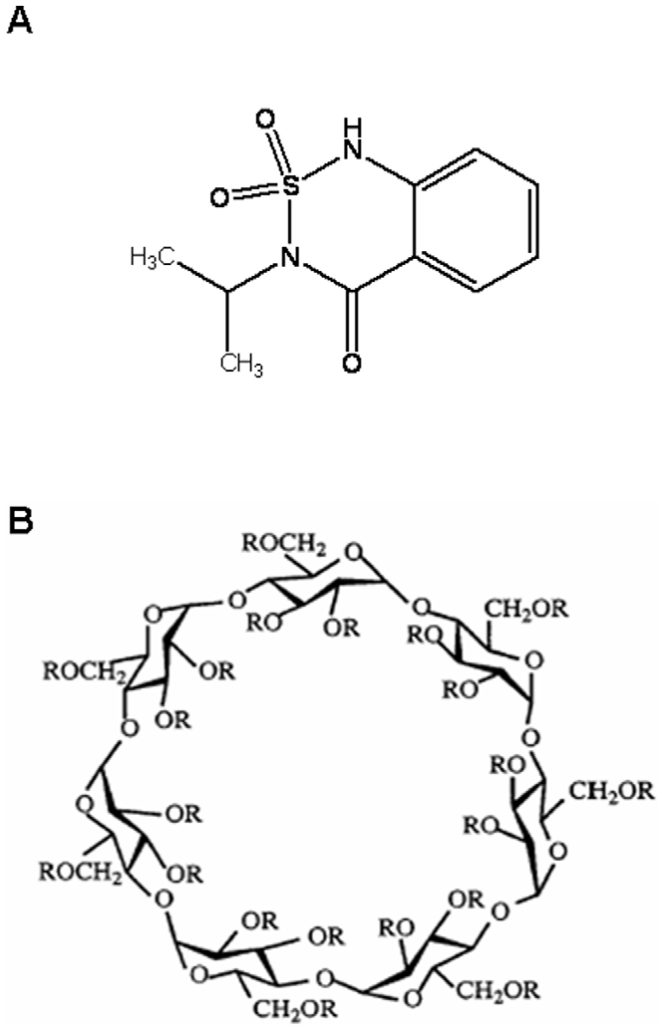
Molecular structures. Bentazon (A) and CD derivative (B), where R is H, 2-hydroxypropyl (CH_2_CHOHCH_3_) or sulfobutylether ((CH_2_)_4_ SO_3_ Na) for βCD, HPCD and SBECD, respectively.

Herbicides are applied to control or kill undesired plants, enabling greater crop productivity and promoting the mechanization of the agricultural production [12]. Bentazon (Btz) 3-(1-methylethyl)-1H-2,1,3-benzothiadiazin-4(3H)-one-2,2-dioxide (Figure 1A) provides effective control of broadleaf weeds and sedges in many crops such as beans, corn, rice, peanuts, peas, asparagus, peppers and peppermint [13]. Btz is a contact post-emergence herbicide that inhibits photosynthesis [14] and is photo-degraded in water with a half-life lower than 24 h [15].

The effectiveness of Btz is often improved by adding adjuvants that increase herbicide activity but also its retention and adsorption; therefore, adjuvants increase phytotoxicity of Btz on many weeds and enhance crop damage [16].

Recently, Ge et al. [17] stated that the herbicide chloropropham increased its solubility and thermal stability when it was in complex with beta-cyclodextrin, βCD, suggesting the use of the latter for herbicide formulations. The utilization of hydroxypropylcyclodextrin (HPCD) in an insecticide formulation has been proposed [18]. In addition, we reported previously the inclusion complex of Btz with βCD and sulfobutylether-βCD (SBECD) in solution, which showed dependence on the functional groups present in the cyclodextrin [19]. General uses of cyclodextrins and their patents were revised by Del Valle [20]. Several commercial products based on CDs are available, as reviewed by Hashimoto [21]. Although an increase of work has been lead towards agrochemical-CD formulations, no large scale commercial applications can be found. Publications and patents have been reviewed by Morillo [22].

In this research we aim to report the preparation and characterization of some inclusion complexes formed by bentazon with native βCD and two derivatives hydroxypropyl-βCD and sulfobutylether-βCD (HPCD and SBECD; Figure 1B). Our main interest is to explore some physicochemical properties of these inclusion complexes with the aim to contribute to the future applications of CDs to solid samples in agriculture field.

## Results and Discussion

### Characterization of inclusion complexes in solution

**Figure 2:**
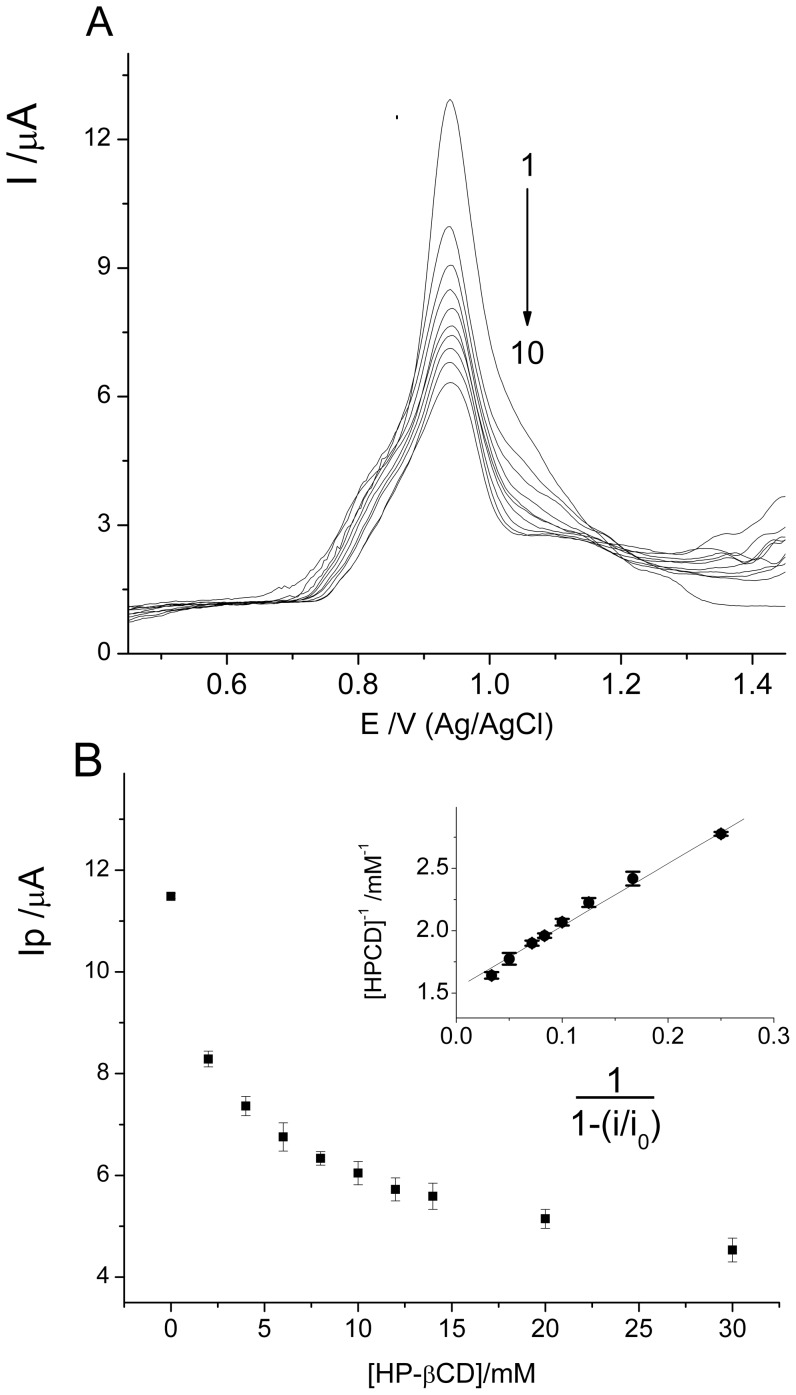
Electrochemical oxidation of bentazon. (A) Differential pulse voltammetry curves for 5.0×10^−4^ M Btz in buffer phosphate pH 6.0 in the absence and presence of different HPCD concentrations. Curves 1–10: 0, 2, 4, 6, 8, 10, 12, 14, 20 and 30 mM HPCD; (B) Current dependence on the concentration of HPCD for bentazon in buffer phosphate pH 6.0. Current values obtained from DPV measurements. Inset: Plot of 1/[CD] versus 1/(1-I/I_0_) for bentazon in buffer phosphate pH 6.0.

**Figure 3:**
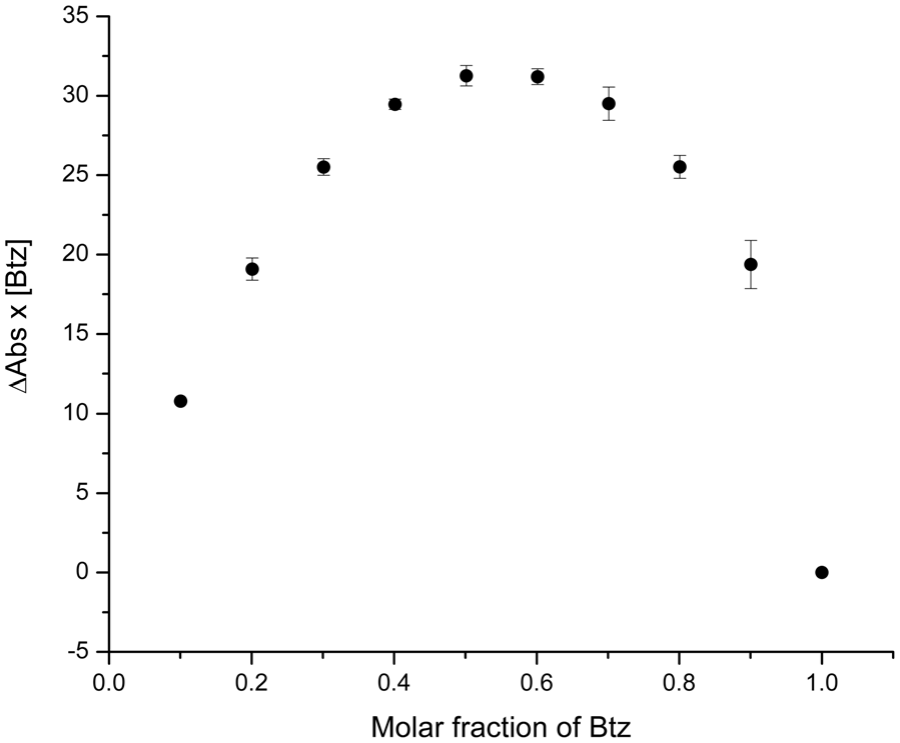
Determination of the stoichiometry of the complex. Continuous variation plot (Job plot) of the absorption change in dependence of the bentazon mole fraction.

The electrochemical behavior of Btz at glassy carbon electrode in aqueous solution in presence of two different cyclodextrin has been previously studied by our research group [19]. The presence of electroactive functionality permits us to use this technique to study the incorporation of Btz into the cyclodextrin cavity. In the case of HPCD, the effect observed is similar to previous results informed, showing a decrease in the current intensity with increasing HPCD concentration (Figure 2A). This decrease of the current (Figure 2B) is associated to the change in apparent diffusion coefficient of the complex compared with the bentazon-free form [19]. The association constant (K_a_) for the complex Btz/HPCD was obtained using the equation described for current titration (Equation 2, Materials and Methods). A linear relationship was obtained in 1/CD vs 1/(1-i/i_0_) plot (Figure 2B, inset). The value for the association constant (K_a_) obtained by DPV was 244±19 M^−1^. This value is between those obtained for βCD and SBECD (118±20 and 317±25 M^−1^, respectively [19]). The K_a_ value for Btz/HPCD inclusion complex differs from that obtained by Porini and Escandar using fluorescence emission [23]. However, the difference is not very significant taking into account that two different methods were used, and also that the association constants are practically of the same order (log K_a_ are 2.3 and 2.1 obtained by DPV and fluorescence, respectively). An association constant value using HPCD higher than that obtained using βCD was expected considering that the polarity of the substituents of the host could contribute to the complex formation. Thus a less polar group like 2-hydroxypropyl can be more favourable to the formation of the complex [23]. In spite of electrochemical results would indicate that a 1∶1 inclusion complex was formed, we have verified the stoichiometry of the inclusion complex obtaining the Job plot (Figure 3). As shown, the maximum value is reached when the mole fraction is 0.5, indicating a 1∶1 stoichiometry.

It is interesting to note that no change in peak potential is observed with HPCD. Previously, we have found different effect of CD on peak potential which could indicate a different insertion of the guest molecules (a change close to 45 mV was observed for a nitro compound indicating that nitro group probably was located inside the cavity) [24]. Thus, no change in peak potential of oxidation of Btz suggests that the electroactive moiety of the molecule is located outside the cavity, consequently the benzene ring would be inside the cavity of cyclodextrin. This possible structure is in agreement with the structure recently proposed by Porini and Escandar [23]. We also have informed a slight shift in a positive direction using βCD and SBECD (a change lower than 20 mV was observed) [19]. This slight shift observed with other CDs, is not enough to propose a different structure. Additional information would be neccesary to verify the structures of the complexes.

### Characterization of solid inclusion complexes

**Figure 4:**
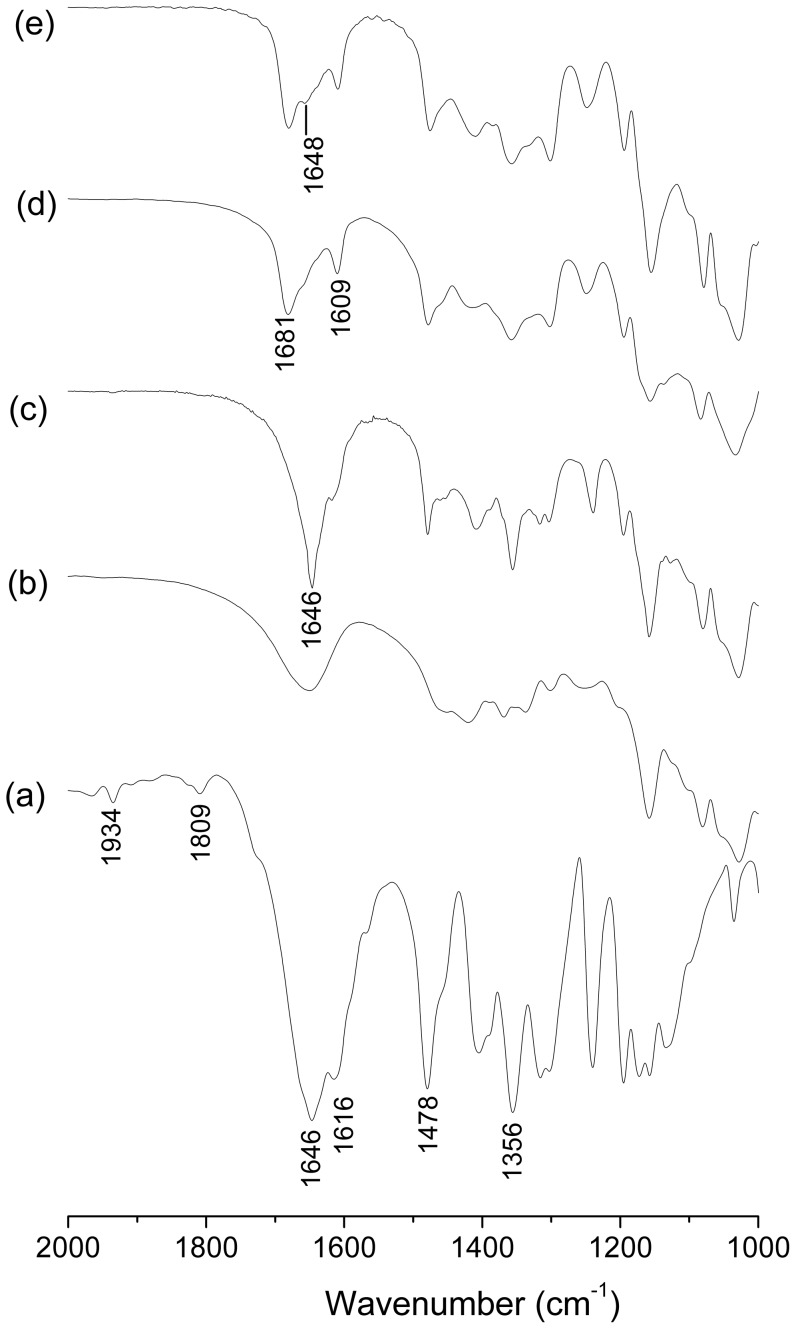
FT-IR spectra. (a) Bentazon, (b) βCD, (c) Btz-βCD (PM), (d) Btz/βCD (KN), (e) Btz/βCD (FD).

**Figure 5:**
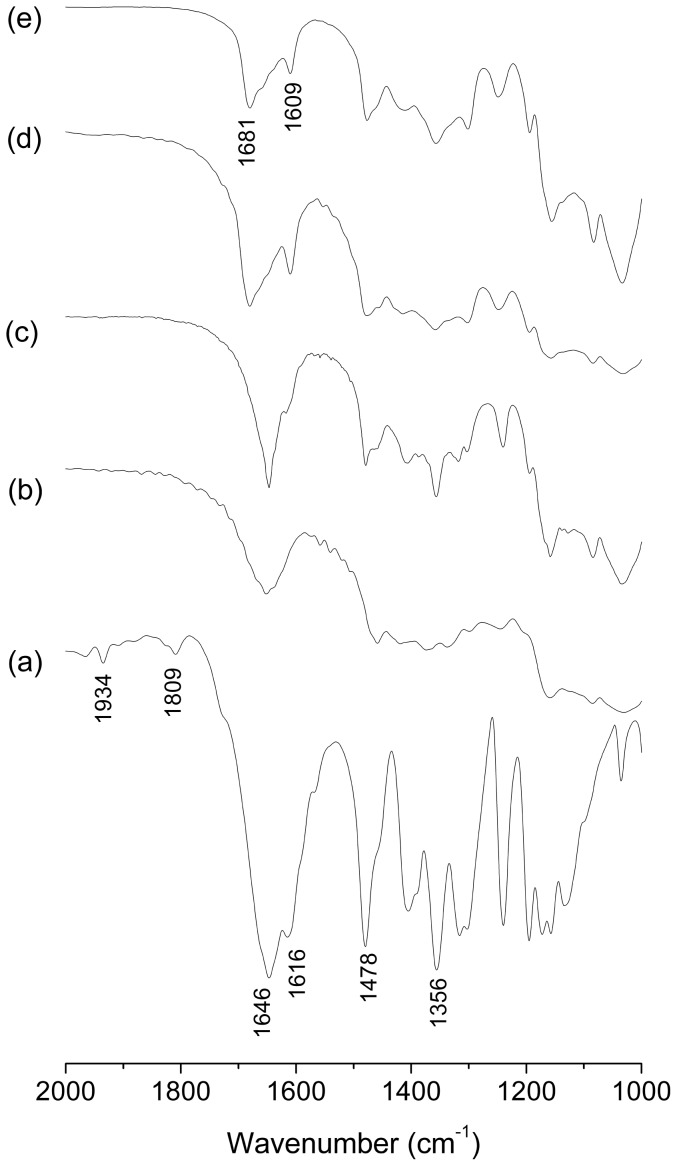
FT-IR spectra. (a) Bentazon, (b) HPCD, (c) Btz-HPCD (PM), (d) Btz/HPCD (KN), (e) Btz/HPCD (FD).

**Figure 6:**
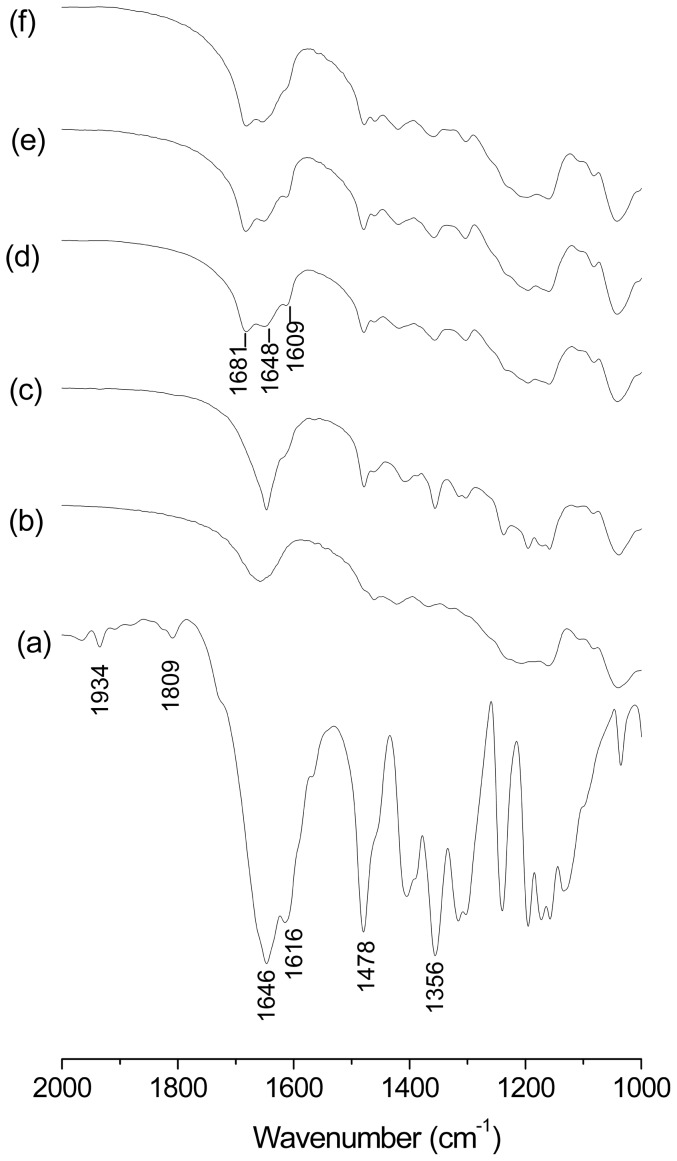
FT-IR spectra. (a) Bentazon, (b) SBECD, (c) Btz-SBECD (PM), (d) Btz/SBECD (KN), (e) Btz/SBECD (FD, 1∶1), (f) Btz/SBECD (FD, 1∶2).

Solid inclusion complexes (1∶1) were prepared by kneading (KN) and freeze-drying (FD) method. Native βCD and two derivatives were used. FT-IR analysis was made to verify the inclusion of the pesticide in the CDs. We have found that this technique is effective in these cases because the principal bands of Btz were not masked by bands from the CDs For the inclusion complexes and the physical mixtures (PM) the bands of Btz are overlapped by the bands of CDs in the range of 3800–2500 cm^−1^, thus these bands are not considered. The results of FTIR spectroscopy are shown in figures 4, 5 and 6. The spectrum of Btz is shown in all of the figures for comparison, and the band assignments are shown in Table 1. The peaks in 1934 and 1809 cm^−1^, corresponding to the bending vibration in the aromatic ring of pure Btz, are not present in the inclusion complexes and physical mixtures. On the other hand, two bands of Btz [25] (1478 and 1356 cm^−1^ corresponding to the C-H bending of the methyl group and S = O stretching vibration in sulfone group, respectively) are visible in the inclusion complexes as well as the physical mixtures, which confirm its presence in the sample. The IR spectra of βCD, HPCD and SBECD showed their characteristic bands in agreement with the previously reported literature [2], [26], [27].

**Table 1:** FT-IR spectrum assignments for Btz.

Wavenumber (cm^−1^)	Assignment
3180	N-H stretching vibration in secondary amine
2981, 2875	C-H stretching vibration in methyl groups
1934, 1809	C-H bending vibration for aromatic ring
1646	C = O stretching vibration in tertiary amide
1616	N-H bending vibration in secondary amine
1478, 1450	C-H bending vibration in methyl groups
1356, 1158	S = O stretching vibration in sulfone group

As can be seen, in the spectrum of the inclusion complex Btz/βCD prepared by kneading (Figure 4d) and Btz/HPCD prepared by kneading (Figure 5d) or freeze-drying (Figure 5e), a peak in 1681 cm^−1^ originally positioned in 1646 cm^−1^ in the spectrum of Btz was observed. This shift shows the disruption of the interaction between molecules of Btz (N-H—O = C), indicating that the herbicide is entrapped into the host cavities as a monomer [25]. The band associated to the bending of N-H (1616 cm^−1^) in the spectrum of Btz, shifts to a lower frequency (1609 cm^−1^) due to the formation of a hydrogen bond. These bands are not present in the physical mixture confirming the formation of an inclusion complex in solid state. Although a small and broad signal in 1648 cm^−1^ is observed for all the CDs, it is clear that there is no overlapping with the bands attributed to the inclusion complexes.

In the other cases (Figure 4e, 6d and 6e) a small band about 1646 cm^−1^ is also present indicating that a mixture of free molecules of bentazon and inclusion complex was obtained. Although Btz/SBECD showed the highest association constant, the solid product was a mixture, even if another ratio (1∶2, Btz:SBECD) was tested (Figure 6f).

**Figure 7:**
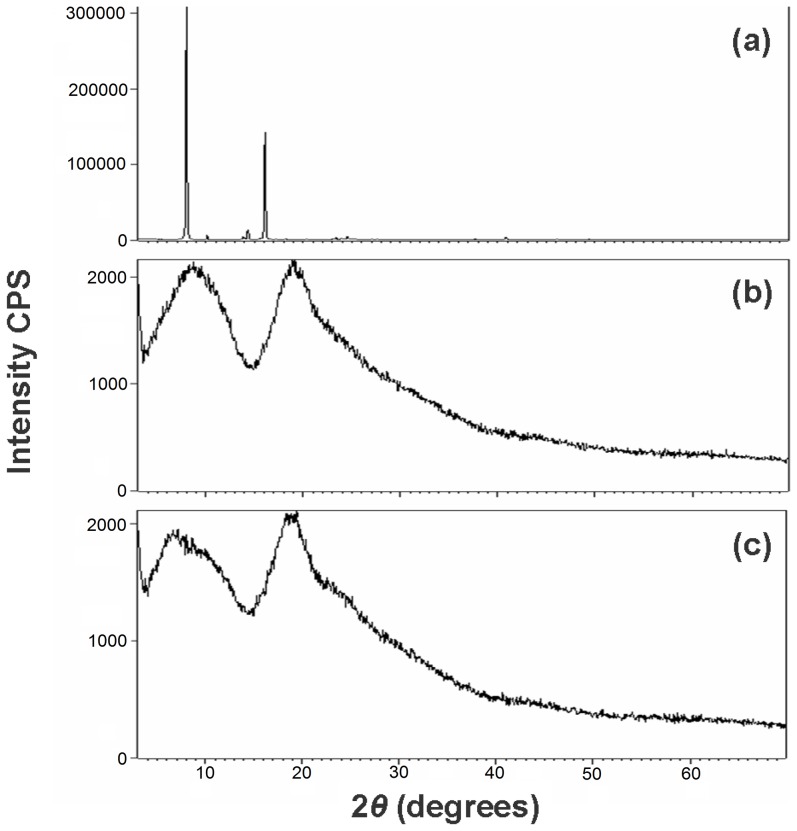
XRD patterns. (A) Bentazon, (B) HPCD and (C) Btz/HPCD inclusion complex (KN).

**Figure 8:**
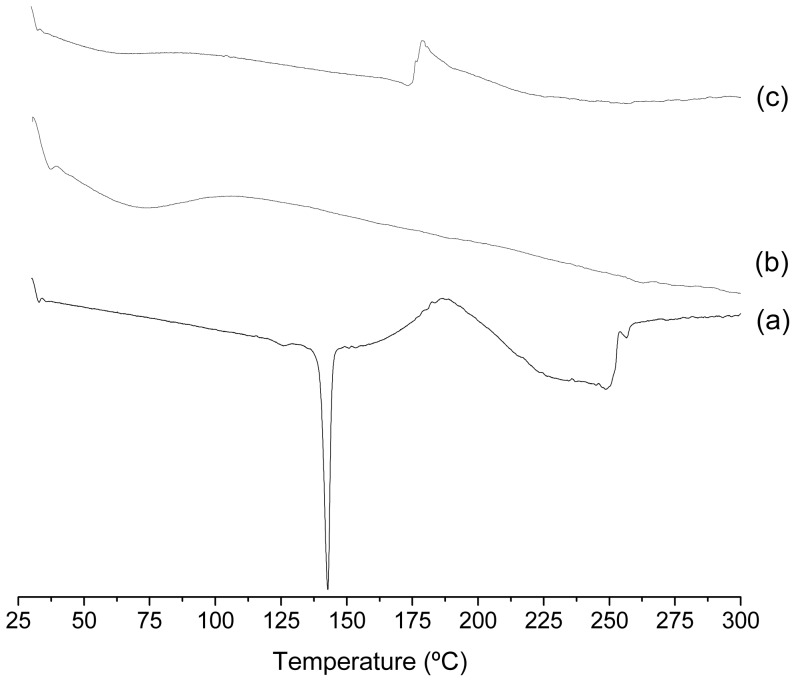
Thermal stability of free molecules and the inclusion complex. DTA thermogram of (a) bentazon, (b) HPCD and (c) Btz/HPCD inclusion complex (KN).

The formation of inclusion complex has been also checked using both, X-ray diffraction (XRD) and differential thermal analysis. The results of the complex Btz/HPCD are shown in figures 7 and 8. The pattern for pure Btz shows its high crystallinity (Figure 7a), with strong and well defined peaks at 10.95 Å and 5.47 Å. The pattern corresponding to HPCD shows its amorphous character (Figure 7b), with two wide bands at approximately 9.95 Å and 4.52 Å. The kneaded complex exhibits no Btz diffraction peaks (Figure 7c), but the new pattern shows the 9.95 Å diffraction of HPCD as a small shoulder over a new band with a maximum at 13.03 Å, suggesting that a new complex has probably been formed but with an amorphous state. The above results support that Btz and HPCD form true inclusion complexes in solid state, with amorphous character when kneading both components. Similar results were obtained for the complex prepared by freeze-drying method (not shown). Finally, differential thermal analysis (DTA) allowed us to determined differences between the thermal stability of the pesticide in a free-form and in the form of an inclusion complex. The thermo-analysis curves obtained for the isolated host, guest and the complex can be seen in figure 8 and data are summarized in Table 2. HPCD shows a broad endothermic peak between 50–100°C corresponding to the process of dehydration. Otherwise, Btz shows an endothermic peak between 130–140°C which corresponds to its melting point and a broad exothermic peak between 170–200°C related with its decomposition [28]. After the inclusion complex is formed, a minor endothermic peak around 60°C is associated to the dehydration of the cyclodextrin. This peak is smaller than the observed for HPCD due to the exchange of Btz for the water molecules located into the cavity of the cyclodextrin. On the other hand, the peak of the melting point of pure Btz disappears, which implies that Btz is included into the cyclodextrin cavity and decompose when inclusion complex does. The exothermic peak around 180°C is associated to the decomposition of the inclusion complex and its enthalpy value is lower than pure Btz. Moreover, the decomposition of the inclusion complex occurs at lower temperatures (about 10°C less), indicating that the complex is slightly less stable at high temperatures.

**Table 2:** Thermal decomposition temperature obtained by DTA.

Sample	Temperature range (°C)	Temperature Peak (°C)	Enthalpy (J g^−1^)	Process
HPCD	40–110	70.4	95.0	Dehydration
Btz	130–140	139.5	184.0	Melting point
	170–200	184.9	−530.0	Decomposition
Inclusion Complex	40–110	65.0	64.5	Dehydration
	170–200	176.9	−156.0	Decomposition

### Photodegradation studies

**Figure 9:**
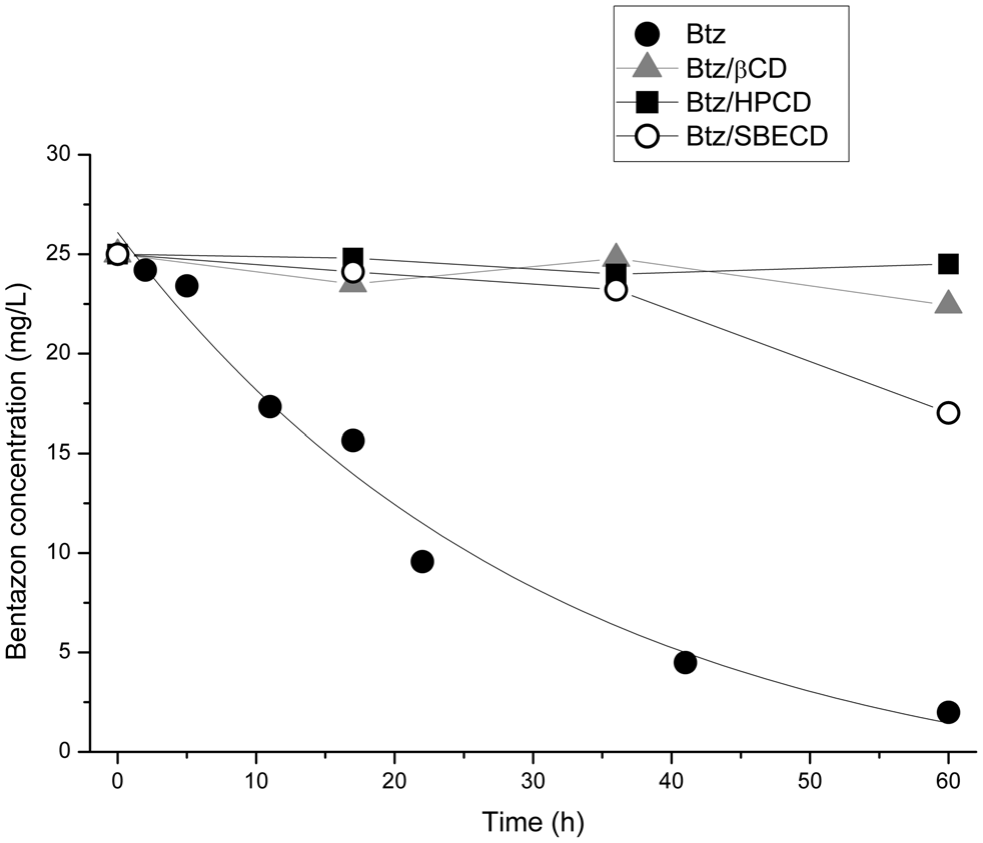
Photodegradation of bentazon. Btz photodegradation profiles in distilled water and in the presence of different cyclodextrins. Bentazon (•), Btz/βCD (▴), Btz/SBECD (○), and Btz/HPCD (▪).

Previous to the photochemical experiments carried out with Btz in solution, photodegradation of solid Btz was also studied for a further study of its photodegradation in the solid complexes with cyclodextrins, but it reached only about 3% after 40 h of exposition. It indicates that the possibility of Btz photodegradation when applied as formulation will be higher in the soil solution than in solid state. According to the formation constants of its complexes in solution with βCD, HPCD and SBECD (118, 244 and 317 M^−1^, respectively), more than 90% of Btz was forming complexes with the cyclodextrins; therefore we decided to study the photodegradation of Btz and their complexes in solution. The photodegradation of Btz in aqueous solution is shown in figure 9. The data were plotted as the concentration of remaining herbicide versus irradiation time. Btz photodegradation profile followed an exponential decay showing that it was clearly affected by photoirradiation in solution, remaining only about 2 mg L^−1^ of Btz after 60 hours. On the contrary, no disappearance of Btz was detected in the dark experiments (not shown), demonstrating that the disappearance of the herbicide was only due to photodegradation, permitting to exclude other phenomena such as volatilization or thermal degradation. Simple first-order kinetics (Eq. 1) was found to be the best descriptor for the experimental data.
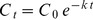
(1)where *C_t_* is the concentration of pesticide remaining in solution (mg L^−1^) at time *t* (hours), *C*_0_ is the initial concentration of pesticide (mg L^−1^) and *k* is the rate of degradation (hours^−1^). The linearized form of Eq. 1 was used to calculate the degradation constants (k) and the time required for 50% disappearance (DT_50_) of Btz, with values of 3.84×10^−2^ h^−1^ and 17.9 h, respectively.

There is a relative controversy among the values of DT_50_ for Btz photodegradation in distilled water in the literature, and also with the value obtained in our paper. Peschka et al. [29], using experimental conditions similar to ours, observed complete photodegradation and formation of SO_3_-bentazon after 13.4 h in pure water, with a DT_50_ about 4 h. Davezza et al. [30] obtained the complete degradation of Btz after 60 min, and its DT_50_ at about 15 min, when using a Xenon lamp of 1500 W m^−2^ and pyrex glass containers instead of quartz ones.

The irradiation of Btz in aqueous solution in the presence of the three cyclodextrins showed no disappearance of the herbicide along the course of irradiation (Figure 9), demonstrating the high photoprotective effect of these cyclodextrins. However, a loss of the herbicide of 32% is observed after 60 h in the case of the complex Btz-SBECD. This loss was not due to Btz photodegradation, because the same loss was also observed in the corresponding dark control, while those corresponding to the other Btz complexes did not show any loss. It indicates that the presence of SBECD facilitated its decomposition after many hours of irradiation and could be probably related to the increased temperature inside the quartz flasks during photochemical experiments (about 60°C).

Although few studies about the photocatalytic effect of CDs on pesticides in aqueous solution have been reported previously, pesticides can exhibit an increase or decrease in the intensity of light absorption when included in the cavity of a cyclodextrin. Zeng et al. [31], concluded that several cyclodextrins acted like photocatalysts in the degradation of the pesticide methyl-parathion, and the same effect occurred when the herbicide norflurazon was irradiated in aqueous solutions in the presence of five different cyclodextrins [32]. However, Kamiya and Nakamura [33] observed that some cyclodextrins promoted the photodegradation of parathion and paraoxon, while others inhibited it. Hanna et al. [34] observed the inhibition effect of β-CD on the photocatalytic degradation of pentachlorophenol in distilled water, and also Orgoványi et al. [35] reported an inhibition of the photodegradation of the insecticide cypermethrin by complexation with two methylated CDs (RAMEB and DIMEB).

### Studies of inclusion complexes in solution

**Figure 10:**
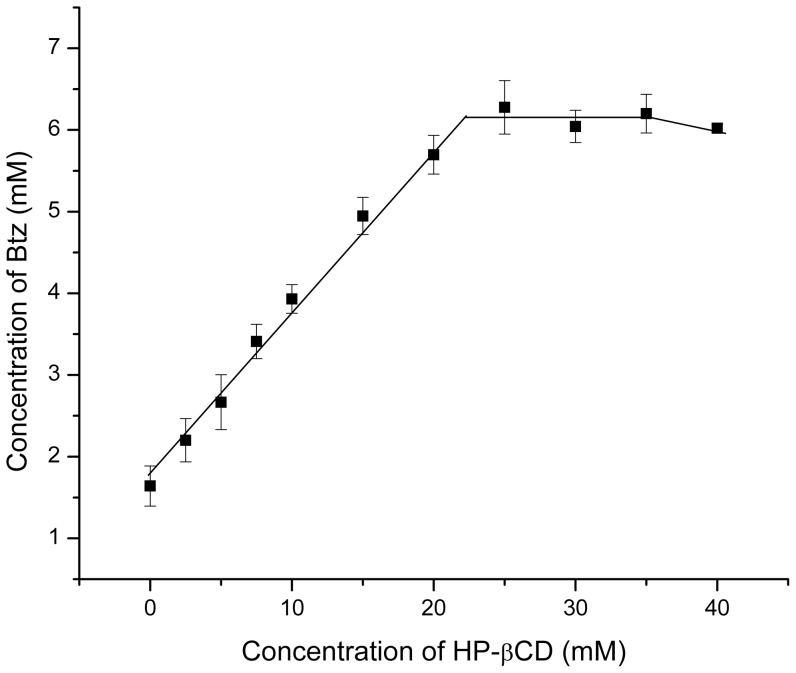
Phase solubility diagram. Concentration of bentazon against increasing concentrations of HPCD.

The phase solubility diagram of Btz in the presence of HPCD is shown in figure 10. Btz showed a Bs type diagram where the solubility of Btz increased linearly with HPCD concentrations up to 22 mM. Then, there is no additional increase in solubility of Btz. Under the experimental conditions used in this experiment, the solubilization efficiency of HPCD, defined here as the increment of the apparent solubility of Btz at the maximum solubility reached with respect to Btz solubility in the absence of HPCD, was about 3.75. HPCD solubilization efficiency is higher than that obtained in presence of βCD although lower than SBECD [19]. This result correlates well with the tendency observed for the association constants of the inclusion complexes under study.

**Figure 11:**
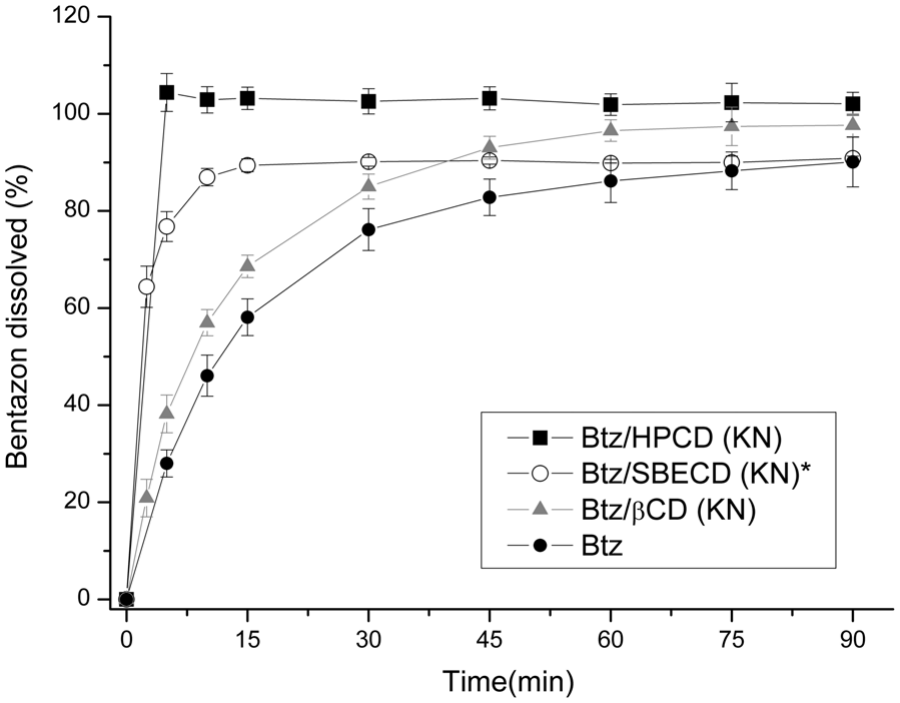
Dissolution test. Dissolution profiles in water of bentazon (•), Btz/βCD (KN) (▴), Btz/SBECD (KN)* (○), and Btz/HPCD (KN) (▪). (*: inclusion complex and free bentazon mixture).

The dissolution studies are shown in figure 11. A faster dissolution rate for inclusion complex with HPCD with respect to the free Btz can be seen, reaching a plateau in a very short time. Btz is completely dissolved before 5 min compared to only 28% of free Btz for the same time and the dissolution of the herbicide (95% dissolved) is reached after 1.5 h. It is shown that no better results were obtained using βCD and SBECD. Although a higher solubility of Btz is reached using SBECD [19], the result obtained in the dissolution test is lower than HPCD probably because the inclusion complex of Btz/SBECD is actually a mixture of both, inclusion complex and free Btz.

The increase in the dissolution of the herbicide is due to the formation of the inclusion complex with CDs. According to Dua et al. [36], the increase in dissolution rate is caused by the effect of CD on lowering the surface tension. On this way, we have measured the surface tension of free Btz, CDs and inclusion complexes solutions. Also, physical mixtures of them were tested for comparison. Results are shown in table 3. No changes in surface tension of water are observed in presence of Btz, βCD or SBECD. However, the surface tension of water decreased to 65.3±0.2 mN/m in presence of HPCD. This value is in agreement to those reported by Leclercq [37] and Nogueiras-Nieto [38], which pointed out that some modified CDs provoke a decrease in surface tension in a different way compared from typical surfactants. We have found that although there was no change in surface tension of Btz or βCD solutions, the inclusion complex showed a lower value. Also, a slight decrease is observed in physical mixtures indicating that some of the complex is being formed.

**Table 3:** Surface tension (γ) of water and aqueous solutions of systems under study.

	γ/mN m^−1^
Water	70.8±0.1
Btz	70.8±0.1
βCD	70.8±0.1
SBECD	70.8±0.1
HPCD	65.3±0.2
Btz- βCD (PM)	66.5±0.4
Btz/βCD (KN)	61.8±0.1
Btz/βCD (FD)*	66.1±0.2
Btz-SBECD (PM)	68.2±0.2
Btz/SBED (KN)*	68.0±0.1
Btz/SBED (FD)*	68.0±0.1
Btz-HPCD (PM)	64.8±0.2
Btz/HPCD (KN)	63.1±0.2
Btz/HPCD (FD)	59.9±0.1

KN (kneading) and FD (freeze drying) indicates methods of preparation of inclusion complexes; PM (physical mixtures) of bentazon (Btz) with different CDs (βCD, SBECD, HPCD).

* : inclusion complex and free bentazon mixture.

The lowest value was obtained with Btz/HPCD inclusion complex. The change observed would indicate that the Btz molecules are interacting with CDs to form inclusion complexes that have surface activity, therefore are adsorbed at the interface which leads a surface excess slightly higher than Btz or even CDs themselves. It is expected that this effect permit an increasing of the contact of the herbicide on the leaf surface after application. A slightly lower surface tension of the Btz/HPCD complex prepared by freeze drying method compared to that prepared by the kneading method is seen in table 3. It is probable that the solid produced by both two different methods exhibits different physical characteristics in solid state, where particle size and crystalline structure of the product, among other factors, could be affecting the wettability and dissolution, and therefore, surface tension. Thus, for example, a reduction in particle size could provoke an increase in wettability and dissolution and, hence lower effect on surface tension. Although the lowest value was obtained for complex prepared by freeze-drying method, the time necessary to obtain such solid inclusion complex is higher than kneading method.

### Conclusions

In this work, the inclusion complexes of Btz with three different CDs were prepared. Two different methods were carried out: kneading and freeze-drying. Host–guest ratio 1∶1 was determined by electrochemical technique and Job Plot. FTIR was used in all the cases to verify the formation of the solid inclusion complexes. DTA and XRD also were used to confirm the formation of an inclusion complex. Solid complexes were obtained for Btz/HPCD independently of the processing method. Besides, solid inclusion complex of Btz/βCD was achieved by kneading; In other cases, mixtures where free bentazon was present together with complex were obtained for Btz/βCD prepared by freeze-drying and complexes using SBECD. We showed that the formation of inclusion complexes with the three CDs used greatly increased the photostability of Btz in aqueous solutions. Besides, CDs improved slightly the solubility of Btz, increased the dissolution rate and decreased the surface tension. Thus, solid complexes with CDs can be obtained by kneading or freeze-drying (depending on the CD) and improved physicochemical properties are observed as compared with Btz. In this work, better properties are obtained for Btz/HPCD complex. Therefore, it is expected that the formation of inclusion complexes with CDs may to improve the properties of Btz formulations, increasing the herbicide effectiveness for future applications.

## Materials and Methods

### Materials

All reagents were of analytical grade and used without prior purification. Bentazon (Btz) was supplied by SIGMA-Aldrich (PS-1011, SUPELCO, Bellefonte, PA, USA). βCD was purchased from Calbiochem (A Brand of EMD Biosciences, Inc. La Jolla, CA. An Affiliate of Merck KGaA, Darmstadt, Germany) 2-Hydroxypropyl-beta-cyclodextrin (HPCD) was supplied by SIGMA-Aldrich (St.Louis, USA). SBECD was obtained from CyDex, Inc. (Lenexa, Kansas). The solutions were prepared with ultrapure water (18.2 MΩ cm) from a Millipore Milli-Q system.

### Preparation and analysis of Btz/HPCD inclusion complexes in solution

An aqueous solution of Btz 5.0×10^−4^ M was divided in flasks of 20 mL, and then HPCD was added in each one in order to obtain samples of different concentrations in the range between 2 and 30 mM. The samples were stirred for 12 h at 25°C and kept in repose for three hours before the measurements.

Differential pulse voltammetry experiments were performed with a potentiostat CHI 440 (CH Instruments). A 10 mL home made cell with a glassy carbon electrode (GCE) (3 mm diam., CHI 104, CH Instruments Inc.) as working electrode was used. A platinum wire and Ag/AgCl (Bioanalytical System) were used as counter and reference electrodes, respectively. The cleanliness of the working electrode was carried out by polishing it with 0.3 and 0.05 µm alumina slurries (Buehler) and then was profusely rinsed with water. Parameters selected: potential scan rate: 20 mV s^−1^, pulse amplitude: 50 mV and pulse width 50 ms.

Voltammetric experiments were carried out by keeping constant the concentration of Btz in 5.0×10^−4^ M, prepared in 0.1 M buffer phosphate (pH 6.0); while the concentration of CD was varied. The current titration equation has been described as follows [39]–[41]:
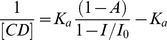
(2)where *K_a_* is the complex association constant, *I_0_* and *I* are the peak currents without and with CD, respectively; [CD] is the molar concentration of CD and A is a constant. The condition for using this equation is that a 1∶1 association complex is formed and CD concentrations are much larger than the total concentration of the guest. All voltammetric experiments were performed after flushing the cell with N_2_ for ten minutes before each run. The temperature was kept constant at 25°C in all experiments.

The stoichiometry of the Btz/HPCD inclusion complex was determined by the equimolar variation method (Job plot) [42] based on the difference in UV-vis absorbance, ΔA (ΔA = A_0_−A), of Btz in presence (A) and absence (A_0_) of HPCD. A series of solutions in which the total concentration of host and guest species was maintained constant and the mole fraction of the guest varied between 0 and 1 were prepared. The concentration used for Btz and HPCD was 2.0×10^−4^ M. The using 1 cm quartz cell and the UV-Vis range used were from 190 to 400 nm.

### Preparation and characterization of solid complexes

Inclusion complexes of Btz with βCD, HPCD and SBECD were prepared by kneading (KN) and freeze-drying (FD) procedures.

The 1∶1 stoichiometric ratio used to prepare solid complexes was obtained from electrochemical results and Job Plot obtained in the present paper for HPCD complex and in a previous paper for BCD and SBECD complexes [19]. Physical mixtures (PM) have also been prepared for comparative purpose, where the Btz and the different CDs (1∶1) were put together without kneading or freezing them. The two different methods employed for the preparation of solid complexes are described:

### Kneading method (KN)

Btz and CDs (1∶1) were mixed in a mortar and kneaded for 45 min. During this process 3 mL of ethanol was added to the mixture to maintain a suitable consistency. The final product was dried at 36°C for 48 h and then was ground to powder and characterized.

### Freeze-drying method (FD)

1∶1 molar ratio of bentazon and CD were dissolved in 60 mL of deionized water and magnetic stirred at 25°C for 24 hours. The solution was freeze-dried in a FreeZone 1.0 Labconco Benchtop Freeze Dry System (Temperature Collector −50°C; Vaccum 0.080 mbar). The time required to dry completely the sample was three days.

FT-IR analyses were carried out on a Bruker IFS 56 FT spectrometer. Spectra were measured with a KBr disk.

Differential thermal analyses were obtained using a differential temperature analyzer (Mettler, FP90 DTA). Samples of about 2–5 mg were encapsulated in aluminum pans and covered with lids which were pierced to permit the gas release during the heat process. The parameters used were a heating rate of 5°C/min over the temperature range from 25 to 300°C.

X-Ray diffraction patterns were obtained using an X-ray diffractometer Siemens model Kristallofex D-5000. The samples were irradiated with Ni-filtered Cu Kα radiation (36 kV, 26 mA), at a scanning rate of 1°/min; chart speed, 1 cm min^−1^ and adequate sensibility, usually 4×10^4^ counts per second.

Photodegradation studies were carried out with a Suntest CPS photoreactor (Heraeus, Germany) equipped with a Xe lamp (500 W/m^2^) with a permanent filter, which selects wavelengths ≥ 290 nm. An aqueous Btz solution (100 mL, 25 mg/L) was irradiated in quartz flasks during 60 hours. The same experiment was carried out in the presence β-CD, HPCD and SBECD at a relation Btz:CD 1∶1 (mol/mol). Dark controls of all experiments were conducted in a similar manner except that the flasks were kept within the reactor covered with aluminum foil. All experiments were carried out in duplicate. Samples were collected from the flasks at different time intervals and the concentration of Btz determined by HPLC under the following conditions: mobile phase, 40∶60 acetonitrile/water containing 0.1% H_3_PO_4_; flow 1 mL min^−1^; chromatographic column, Kromasil C18 (150 mm length×4 mm i.d.) (Teknokroma, Spain); diode array detector (Shimadzu SPD-M10A) at a selected λ of 225 nm. The retention time for Btz under these conditions was 5.2 min.

Phase solubility diagrams were performed in triplicate according to the method carried out previously [19]. The dissolution rate studies were carried out separately in 500 mL deionized water with paddle method using Varian VK 7010 dissolution testing instrument with a Varian VK 750D heater-circulator. Samples containing 30 mg of Btz were sprinkled over the surface of the dissolution media. The stirring speed was 50 rpm and the temperature was maintained at 25±0.1°C. 5 mL aliquots were withdrawn at different time intervals up to 1.5 hours, then were filtered and analyzed by spectrophotometry UV-Visible. Same volume of fresh deionized water was added to maintain the total volume. The amount of Btz was estimated by measuring the absorbance at 333.5 nm. The dissolution experiments were done in triplicate.

Surface tension measurements were performed preparing Btz, HPCD, physical mixture and Btz/HPCD inclusion complex solutions (5×10^−4^ M) in ultrapure water. Surface tension measurements were made in quintuplicate at 25±0.1°C by the Du Noüy Ring method using a tensiometer EasyDyne Krüss.
